# The Function of T Follicular Helper Cells in the Autoimmune Liver Diseases

**DOI:** 10.1155/2020/5679254

**Published:** 2020-11-18

**Authors:** Lin Li, Panyang Xu, Qi Zhou, Jiancheng Xu

**Affiliations:** ^1^Department of Laboratory Medicine, First Hospital of Jilin University, Changchun 130021, China; ^2^Department of Pediatrics, First Hospital of Jilin University, Changchun 130021, China

## Abstract

T follicular helper (TFH) cells are recognized as a subtype of T cells that are involved in the germinal center formation and B cell development. When dysregulated, TFH cells may represent an important mechanism that contributes to a heightened humoral response and autoantibody production in autoimmune liver diseases (AILDs). TFH cells participate in the immune response associated with AILDs by expressing surface receptors such as programmed cell death protein-1, C-X-C motif chemokine receptor 5, and inducible T cell costimulators, as well as cytokines such as interleukin-21. TFH cells also downregulate chemokine (C-C motif) receptor 7 and promote the dysregulation of the T follicular regulatory/TFH axis. This review highlights the importance of TFH cells in AILDs.

## 1. Introduction

**Figure 1:**
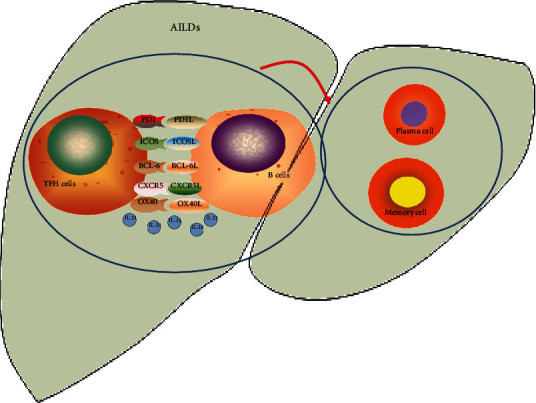
AILDs: autoimmune liver diseases; PD-1: programmed cell death protein-1; CXCR5: CXC chemokine receptor 5; ICOS: inducible T cell costimulator; IL-21: cytokine interleukin- (IL-) 21; BCL-6: B cell lymphoma 6; OX40: tumor necrosis factor receptor. TFH plays a key role in assisting B cells. Autoimmune diseases are characterized by excessive activation of B cells, leading to the production of autoantibodies and attacking their normal tissues. This suggests that TFH cells may have an important influence on the pathogenesis of autoimmune diseases.

Autoimmune liver disease is a group of chronic hepatobiliary inflammatory diseases mediated by autoimmune response, mainly consist of autoimmune hepatitis (AIH), primary biliary cholangitis (PBC), primary sclerosing cholangitis (PSC), and IgG4-related sclerosing cholangitis (IgG4-SC) [1]. T follicular helper (TFH) cells are special subtypes of CD4^+^ T cells that have evolved appropriate mechanisms to induce B cell activation and differentiation into immunoglobulin (Ig-) secreting cells (plasma cells) [2]. In secondary lymphoid tissues, TFH cells have an important influence on the formation of the germinal center (GC) and the development of T cell-dependent B cell responses [3]. One distinctive feature of TFH is that they have the high surface expression of C-X-C motif chemokine receptor 5 (CXCR5), which can induce TFH cells to transfer to the follicular area of B cells expressing CXCL13 (the ligand of CXCR5). TFH cells can also regulate humoral immune response through secretion and expression of various cytokines, including the signal transcription factor B cell lymphoma 6 (BCL-6), programmed cell death protein-1 (PD-1), CD40 ligand, and the cytokine interleukins IL-21, IL-10, and IL-6 [4–6]. Circulating TFH cells can be classified into three different subsets (TFH17, TFH1, and TFH2) in view of the subtype cytokine profiles and effectiveness in supporting B cells [7] (see Figure 1). We also introduce a T cell subtype that is closely related to TFH cells: T follicular regulatory (TFR) cells, which are in the germinal center and have the same phenotypic traits as TFH cells. The differentiation of TFR cells is an extremely complex process. The differentiation of TFR cells was initiated by dendritic cells; development and expansion required the help of B cells, costimulatory signals, such as CD28 and ICOS, and the expression of transcription factors such as BCL-6 [8–12].TFR cells play a negative regulatory role in germinal center reaction. TFR cells may inhibit plasma cells by inhibiting the GC reaction. When plasma cells migrate from secondary lymphoid organs to bone marrow, TFR cells will lose the inhibitory effect on plasma cells [13]. More evidence indicated that the imbalance between circulating TFR and TFH cells might cause the immune system's tolerance disorders and the production of abnormal autoantibodies. These pathogenic mechanisms are considered a crucial development of autoimmune responses [14]. This article will investigate the contribution of TFH cells to the disease pathogenesis in AILDs.

## 2. Autoimmune Hepatitis (AIH)

AIH is a chronic inflammatory liver disease induced by decreased immunological tolerance to liver autoantigens. The clinical features of AIH include the detection of autoantibodies via liver histology, hyperglobulinemia, and lymphocytic plasma infiltration of interface hepatitis [15]. In a prospective population-based study, Lamba et al. found that the incidence of AIH in New Zealand tended to increase annually [16]. However, the reason for the change in the incidence of AIH has not yet been discovered. Evidence in recent years has shown that B cells have an important influence on autoimmune diseases. During the pathogenesis and development of AIH autoimmunity, TFH cells provided the B cells in the GC area with a key signal to produce autoantibodies [17]. Over expanded TFH cells could lead to overreactions of the GC, such as the disordered proliferation of self-reactive B cells, excessive differentiation of long-lived plasma cells, and the mass secretion of high-affinity pathogenic autoantibodies. The pathological enrichment of TFH cells could be critical for the survival of homologous self-reactive B cells and the tolerance check of escaping the GC area [3]. These observations indicated the contribution of TFH cells in autoimmune hepatitis, and TFH cells are mainly affected by three substances: interleukin-21, programmed cell death protein-1, and T follicular regulatory cells.

### 2.1. Interleukin-21 (IL-21)

TFH cells express high levels of IL-21 that influence B cell differentiation and antibody production [3]. IL-21 belongs to the type I cytokine family; type I cytokines have massive effects on the immune system, including B cell activation in the GC of the secondary lymphoid organs, plasma cell differentiation, and immunoglobulin production [2, 18]. Although the data on IL-21-induced direct antibody-mediated cytotoxicity is limited, the circumstantial evidence shows that IL-21 may mediate the production of autoantibodies and has an important influence on the pathogenesis of AIH. Ma et al. found that patients with new-onset AIH had higher serum IL-21 levels, which was accompanied by plasma cells, activated B cells, TFH cells, and serum immunoglobulins [19]. Subsequently, researchers explored whether TFH cells participated in AIH autoimmunity through the elevated secretion of IL-21. Using coculture experiments, Morita et al. reported that TFH2 and TFH17, but not TFH1, induced naive B cells to produce immunoglobulin by secreting IL-21 [7]. Meanwhile, in the mouse model of AIH, blocking IL-21 secretion effectively inhibited the production of TFH cells and prevented the development of AIH in the mice [20, 21]. Abe et al. found that serum levels of IL-21 were prominently higher among AIH patients contrasted to nonsevere liver disease patients; this increase was positively correlated with necrotizing inflammatory activity [18]. Serum IL-21 levels are expected to be an indicator for predicting the evolution of necrotizing inflammatory activity in liver histology, providing important evidences regarding AIH diagnosis, and identifying necessary therapeutic targets [18]. In summary, researchers found that TFH cells were involved in the pathogenesis of AIH by secreting IL-21, and the serum concentration of IL-21 in severe AIH patients is significantly increased through animal and human studies. These studies indirectly prove that TFH cells involve in the pathogenesis of AIH by secreting IL-21 cytokines, which has severity with the disease.

### 2.2. Programmed Cell Death Protein-1 (PD-1)

The TFH cell surface receptor PD-1 inhibits the adaptive immune response by binding to its ligand, programmed cell death ligand- (PDL-) 1, or PDL-2 [22]. The role of chemokine (C-C motif) receptor 7 (CCR7) is the opposite of PD-1 and promotes a variety of adaptive immune functions [23]. The researchers found that there were CCR7^−^PD-1^+^ TFH cell subtypes in the peripheral blood of AIH patients. Quantifying CCR7^−^PD-1^+^ TFH cells in the peripheral blood of AIH patients might be used to the auxiliary diagnosis of AIH, which thus also confirming that TFH cells participated in the pathogenesis of AIH [24, 25].

The chemokine receptor CXCR5, the nuclear transcriptional repressor BCL-6 [12], and the surface receptor-inducible T cell costimulator (ICOS) [3] may also control TFH cell transcription. However, whether TFH cells participate in the pathogenesis of AIH through CXCR5 and ICOS has not yet been confirmed, and more basic researches are needed to support this hypothesis.

### 2.3. T Follicular Regulatory (TFR) Cells

The dysregulation of TFR and TFH cells is closely related to the pathological mechanisms of autoimmune diseases. Liang et al. closely followed the relationship between the dysregulation of TFR and TFH cells and the pathogenesis of AIH. Studies found that in AIH patients, TFR cell counts were negatively related to TFH cell numbers and IL-21 levels but positively related to the inhibitory factors IL-10 and TGF-*β*1. Collectively, the decrease of CTLA-4 and TFR-related factor IL-10/TGF-*β*1 and the increase of PD-1/ICOS in AIH patients lead to the decrease of TFR cells, while the increase of TFH-related factor IL-21 increases the number of TFH cells and the decrease of TFR/TFH ratio, thus promoting the differentiation of B cells and the production of immunoglobulin.

## 3. Primary Biliary Cholangitis (PBC)

PBC, which is once described as primary biliary cirrhosis, is an autoimmune disease that occurs more frequently among women. PBC is characterized by chronic cholestasis, which leads to the injury of small hepatic bile ducts, inflammation, and eventually progressive fibrosis [15]. Because of genetic risk factors and decreased environmental tolerance, PBC is often correlated with autoimmune diseases (such as chronic thyroiditis and Sjogren's syndrome). Liver transplantation is often required because of liver failure; however, PBS often reoccurs after liver transplantation [26]. Studies have shown that the pathogenesis of PBC is closely related to TFH cells. The following sections will focus on the relationships between TFH cells and PBC. Similarly, in PBS, TFH cell count was associated with TFR cells. Besides, CXCR5 and other cytokines affected TFH cell levels.

### 3.1. CXCR5

CXCR5 is a TFH-related factor that is also considered a risk factor for PBC [27]. Circulating CD4^+^CXCR5^+^ TFH cells are a subtype of memory TFH cells that may exist in the blood for a long time [7]. When antigenic stimulation occurs, these memory TFH cell subsets can quickly transform into TFH cells to promote the GC response [28]. When the production of circulating TFH cells is out of control, it reflects GC imbalance and causes an abnormal elevation in the number of autoreactive B cells as well as the production of pathogenic autoantibodies. At this time, clinical symptoms often appear. When the immune response continues, irreversible tissue damage will eventually occur [29]. Wang et al. detected circulating of CD4^+^CXCR5^+^ TFH cells in the peripheral blood of PBC patients [6]. They also found that elevated numbers of TFH in PBC patients were correlated with B cell activation, disease severity, and response to ursodeoxycholic acid treatment [6]. The above reports give us a deeper understanding of the immune pathogenesis of PBC. CD4^+^CXCR5^+^ TFH cells may become a marker for monitoring the effect of treatment in PBC patients. However, it is still unknown why high numbers of CD4^+^CXCR5^+^ TFH cells are positively correlated to the disease severity in PBC patients [30]. Researches are still needed to be explored for its fundamental mechanism.

### 3.2. Other Cytokines

Wang et al. demonstrated that elevated numbers of circulating ICOS+ TFH, IL-21+ TFH [31], and PD-1+ TFH2 cells [32] might be found in PBC patients. Adam et al. also confirmed that PBC patients had significantly higher numbers of CXCR5+PD-1+ and CD4+ TFH cells [33]. Additionally, the activation marker OX40 and inducible T cell costimulatory factors were highly expressed in PBC and were related to the titers of antimitochondrial antibody M2 and IgM. When PBC patients developed cirrhosis, its serological level was detected that there was a significant upward trend [33].

Significant increases in concentrations of OX40, CXCR5, PD-1, ICOS, and IL-21 have been observed in PBC patients. These previous studies showed that TFH cells were closely associated with the pathogenesis of PBC and provided an important tool for the diagnosis and treatment of PBC. However, these molecular mechanisms are not yet fully understood; more researches are still needed.

### 3.3. TFR Cells

The roles of dysregulated TFR and TFH cells in the immune systems of PBC patients are controversial. Zheng et al. pointed out that the serum TFR/TFH ratio of PBC patients was remarkably lower than that of the normal control group [34]. This study also indirectly proved that the dysregulation of the circulating TFR/TFH ratio was involved in the pathogenesis of PBC. Therefore, this ratio might be used as a serological marker for developing new therapies and evaluated therapeutic efficacy in PBC patients [34]. However, Adam et al. found that although PBC patients showed a higher count of TFR cells, the TFH/TFR ratio was not remarkably different from that of healthy people [33]. Therefore, more researches are required to confirm whether there is a significant change in the circulating TFR/TFH ratio in PBC.

## 4. Primary Sclerosing Cholangitis (PSC)

PSC is an uncommon illness whose distinguishing features are multifocal bile duct strictures and progressive liver disease [35]. There are no clinical manifestations during the disease; rather, the pathophysiological mechanism manifests as anterior cholestasis. Subsequently, PSC will develop progressive biliary strictures, which lead to recurrent cholangitis, biliary cirrhosis, and end-stage liver disease [36]. Therapy will not slow the disease progression of this disease. Many patients require liver transplantation, after which there is a risk of recurring disease [36]. Although significant progress has been made in defining the immunological features related to the deficiency of tolerance, there is little information on the biological effects of biliary injury [26]. Furthermore, the pathogenesis of PSC has not been precisely defined. Perinuclear antineutrophil cytoplasmic antibodies may be detected in most PSC patients, which suggest that its pathogenesis may be related to immune disorders. However, various studies indicated that the pathogenesis of PSC did not support its classification as an autoimmune disease. For example, PSC patients had male dominance, and lack of clear autoantigens and immunosuppressants did not affect the disease process [26]. By detecting CXCR5^+^PD-1^+^CD4^+^ TFH cells in patients, Adam et al. found that TFH cells affected the occurrence of PSC to a lesser extent, which supported the above hypothesis [33]. However, there are numerous debates over whether PSC can be regarded as a true autoimmune disease.

## 5. IgG4-Related Sclerosing Cholangitis (IgG4-SC)

IgG4-SC, the biliary manifestation of the systemic fibroinflammatory condition, is featured by the enrichment of IgG4-positive plasma cells and CD4+ T cells in associated tissues [37]. IgG4-SC has been gradually valued because of its high rate of organ dysfunction and failure, high rate of recurrence, and high mortality rate [38]. Although serum concentrations of IgG4 and IgE are elevated in mass PSC patients, they are not enough to be diagnosed and monitored with disease activity [39, 40]. Studies have found that PD-1 may be associated with the proliferation and activation of TFH cells, leading to IgG4-SC.

### 5.1. PD-1

PD-1 is a milestone of cell activation in TFH cells, which is important to the selection of B cells and survival in GCs, along with the transformation of B cells into antibody-secreting cells [7]. Cargill et al. found that circulating and tissue-activated TFH cells were expanded in IgG4-SC, correlated with disease activity, and drove the class switch and proliferation of IgG4-committed B cells. PD-1^+^ TFH2 cells are possible to be a marker of activating disease and a latent target for immunotherapy [32].

Previous studies paid attention to the immune mechanism of the disease found that TFH cells were closely correlated with the immune mechanism of IgG4-SC. However, new literature is still needed to support this argument.

## 6. Conclusion

TFH cells have an important influence on the pathogenesis of AILDs, especially in AIH, PBC, and IgG4-SC. Whether TFH cells participated in the pathogenesis of PSC remains to be discussed (Table 1). In summary, the literature on the contribution of TFH cells in the pathogenesis of AILDS is still in its infancy. The immune system is a complex and well-regulated network. Furthermore, the discovery and correct understanding of TFH cells provide insight into the pathogenesis of autoimmune diseases. TFH cells may be used as a new diagnostic marker and treatment point in the clinic, thus bringing new hope to the treatment of AILDs.

## Figures and Tables

**Table 1:** Influence of follicular helper T cells (TFH) in human and mouse AILDs.

Disease	ICOS	IL-21	PD-1	CXCR5	BCL-6	OX40	TFR/TFH ratio	References	
								Mouse	Human
AIH	-	Pathogenic	Pathogenic	-	-	-	Pathogenic	[20, 21]	[19] [7] [18] [24, 25] [17]
PBC	Pathogenic	Pathogenic	Pathogenic	-	-	Pathogenic	Controversial	-	[6] [31] [32] [33] [34]
PSC	-	-	Controversial	Controversial	-	-	-	-	[33]
IgG4-SC	-	-	Pathogenic	-	-	-	-	-	[32]

AILDs: autoimmune liver diseases; AIH: autoimmune hepatitis; PBC: primary biliary cholangitis; PSC: primary sclerosing cholangitis; IgG4-SC: IgG4-related sclerosing cholangitis; PD-1: programmed cell death protein-1; CXCR5: CXC chemokine receptor 5; ICOS: inducible T cell costimulator; IL-21: cytokine interleukin- (IL-) 21; BCL-6: B cell lymphoma 6; OX40: tumor necrosis factor receptor; TFR: T follicular regulatory.

## References

[1] Liu S. P., Bian Z. H., Zhao Z. B. (2020). Animal models of autoimmune liver diseases: a comprehensive review. *Clinical Reviews in Allergy and Immunology*.

[2] Ma C. S., Deenick E. K. (2014). Human T follicular helper (Tfh) cells and disease. *Immunology and Cell Biology*.

[3] Mesquita D., Cruvinel W. M., Resende L. S. (2016). Follicular helper T cell in immunity and autoimmunity. *Brazilian Journal of Medical and Biological Research*.

[4] Breitfeld D., Ohl L., Kremmer E. (2000). Follicular B helper T cells express CXC chemokine receptor 5, localize to B cell follicles, and support immunoglobulin production. *Journal of Experimental Medicine*.

[5] Gunn M. D., Ngo V. N., Ansel K. M., Ekland E. H., Cyster J. G., Williams L. T. (1998). A B-cell-homing chemokine made in lymphoid follicles activates Burkitt’s lymphoma receptor-1. *Nature*.

[6] Wang L., Sun Y., Zhang Z. (2015). CXCR5+ CD4+ T follicular helper cells participate in the pathogenesis of primary biliary cirrhosis. *Hepatology*.

[7] Morita R., Schmitt N., Bentebibel S. E. (2011). Human blood CXCR5(+)CD4(+) T cells are counterparts of T follicular cells and contain specific subsets that differentially support antibody secretion. *Immunity*.

[8] Sage P. T., Francisco L. M., Carman C. V., Sharpe A. H. (2013). The receptor PD-1 controls follicular regulatory T cells in the lymph nodes and blood. *Nature Immunology*.

[9] Chung Y., Tanaka S., Chu F. (2011). Follicular regulatory T cells expressing Foxp3 and Bcl-6 suppress germinal center reactions. *Nature Medicine*.

[10] Gerner M. Y., Torabi-Parizi P., Germain R. N. (2015). Strategically localized dendritic cells promote rapid T cell responses to lymph-borne particulate antigens. *Immunity*.

[11] Sage P. T., Alvarez D., Godec J., von Andrian U. H., Sharpe A. H. (2014). Circulating T follicular regulatory and helper cells have memory-like properties. *Journal of Clinical Investigation*.

[12] Zhu Y., Zou L., Liu Y. C. (2016). T follicular helper cells, T follicular regulatory cells and autoimmunity. *International Immunology*.

[13] Shlomchik M. J., Weisel F. (2012). Germinal center selection and the development of memory B and plasma cells. *Immunological Reviews*.

[14] Linterman M. A., Pierson W., Lee S. K. (2011). Foxp3+ follicular regulatory T cells control the germinal center response. *Nature Medicine*.

[15] Taylor S. A., Assis D. N., Mack C. L. (2019). The contribution of B cells in autoimmune liver diseases. *Seminars in Liver Disease*.

[16] Lamba M., Hieng N. J., Stedman C. (2020). Trends in incidence of autoimmune liver diseases and increasing incidence of autoimmune hepatitis. *Clinical Gastroenterology and Hepatology*.

[17] Liang M., Liwen Z., Juan D., Yun Z., Yanbo D., Jianping C. (2020). Dysregulated TFR and TFH cells correlate with B-cell differentiation and antibody production in autoimmune hepatitis. *Journal of Cellular and Molecular Medicine*.

[18] Abe K., Takahashi A., Imaizumi H. (2016). Interleukin-21 plays a critical role in the pathogenesis and severity of type I autoimmune hepatitis. *Springerplus*.

[19] Ma L., Qin J., Ji H., Zhao P., Jiang Y. (2014). Tfh and plasma cells are correlated with hypergammaglobulinaemia in patients with autoimmune hepatitis. *Liver International*.

[20] Aoki N., Kido M., Iwamoto S. (2011). Dysregulated generation of follicular helper T cells in the spleen triggers fatal autoimmune hepatitis in mice. *Gastroenterology*.

[21] Ma L., Zhang L. W., Zhuang Y., Ding Y. B., Chen J. P. (2019). Exploration the significance of Tfh and related molecules on C57BL/6 mice model of experimental autoimmune hepatitis. *Journal of Microbiology, Immunology and Infection*.

[22] Mallett G., Laurence A., Amarnath S. (2019). Programmed cell death-1 receptor (PD-1)-mediated regulation of innate lymphoid cells. *International Journal of Molecular Sciences*.

[23] Comerford I., Harata-Lee Y., Bunting M. D., Gregor C., Kara E. E., McColl S. R. (2013). A myriad of functions and complex regulation of the CCR7/CCL19/CCL21 chemokine axis in the adaptive immune system. *Cytokine & Growth Factor Reviews*.

[24] Kimura N., Yamagiwa S., Sugano T. (2018). Possible involvement of chemokine C-C receptor 7(-) programmed cell death-1(+) follicular helper T-cell subset in the pathogenesis of autoimmune hepatitis. *Journal of Gastroenterology and Hepatology*.

[25] Kimura N., Yamagiwa S., Sugano T. (2019). Usefulness of chemokine C-C receptor 7−/programmed cell death‐1+follicular helper T cell subset frequencies in the diagnosis of autoimmune hepatitis. *Hepatology Research*.

[26] Carey E. J., Ali A. H., Lindor K. D. (2015). Primary biliary cirrhosis. *Lancet*.

[27] Mells G. F., Floyd J. A., Morley K. I. (2011). Genome-wide association study identifies 12 new susceptibility loci for primary biliary cirrhosis. *Nature Genetics*.

[28] Papp G., Szabo K., Szekanecz Z., Zeher M. (2014). Follicular helper T cells in autoimmune diseases. *Rheumatology (Oxford)*.

[29] Simpson N., Gatenby P. A., Wilson A. (2010). Expansion of circulating T cells resembling follicular helper T cells is a fixed phenotype that identifies a subset of severe systemic lupus erythematosus. *Arthritis and Rheumatism*.

[30] Zhou Z. Q., Tong D. N., Guan J. (2017). Circulating follicular helper T cells presented distinctively different responses toward bacterial antigens in primary biliary cholangitis. *International Immunopharmacology*.

[31] Wang L., Sun X., Qiu J. (2015). Increased numbers of circulating ICOS(+) follicular helper T and CD38(+) plasma cells in patients with newly diagnosed primary biliary cirrhosis. *Digestive Diseases and Sciences*.

[32] Cargill T., Makuch M., Sadler R. (2019). Activated T-follicular helper 2 cells are associated with disease activity in IgG4-related sclerosing cholangitis and pancreatitis. *Clinical and Translational Gastroenterology*.

[33] Adam L., Zoldan K., Hofmann M. (2018). Follicular T helper cell signatures in primary biliary cholangitis and primary sclerosing cholangitis. *Hepatology Communications*.

[34] Zheng J., Wang T., Zhang L., Cui L. (2017). Dysregulation of circulating Tfr/Tfh ratio in primary biliary cholangitis. *Scandinavian Journal of Immunology*.

[35] Karlsen T. H., Folseraas T., Thorburn D., Vesterhus M. (2017). Primary sclerosing cholangitis - a comprehensive review. *Journal of Hepatology*.

[36] Hirschfield G. M., Karlsen T. H., Lindor K. D., Adams D. H. (2013). Primary sclerosing cholangitis. *Lancet*.

[37] Culver E. L., Chapman R. W. (2016). IgG4-related hepatobiliary disease: an overview. *Nature Reviews. Gastroenterology & Hepatology*.

[38] Huggett M. T., Culver E. L., Kumar M. (2014). Type 1 autoimmune pancreatitis and IgG4-related sclerosing cholangitis is associated with extrapancreatic organ failure, malignancy, and mortality in a prospective UK cohort. *American Journal of Gastroenterology*.

[39] Culver E. L., Sadler R., Bateman A. C. (2017). Increases in IgE, eosinophils, and mast cells can be used in diagnosis and to predict relapse of IgG4-related disease. *Clinical Gastroenterology and Hepatology*.

[40] Culver E. L., Sadler R., Simpson D. (2016). Elevated serum IgG4 levels in diagnosis, treatment response, organ involvement, and relapse in a prospective IgG4-related disease UK cohort. *American Journal of Gastroenterology*.

